# To whom do the results of the multicenter, randomized, controlled INSECT trial (ISRCTN 24023541) apply? - assessment of external validity

**DOI:** 10.1186/1471-2482-12-2

**Published:** 2012-02-08

**Authors:** Lars Fischer, Hanns P Knaebel, Henriette Golcher, Thomas Bruckner, Markus K Diener, Jeannine Bachmann, Markus W Büchler, Christoph M Seiler

**Affiliations:** 1 Department of General, Visceral and Transplantation Surgery, University of Heidelberg, Im Neuenheimer Feld 110, Heidelberg, 69120, Germany; 2 Study Center of the German Surgical Society, University of Heidelberg, Im Neuenheimer Feld 110, Heidelberg, 69120, Germany; 3 Aesculap AG, Am Aesculap-Platz, Tuttlingen. 78532, Germany; 4 Department of Surgery, University of Erlangen, Krankenhausstrasse 12, Erlangen, 91054, Germany; 5 Institute of Medical Biometry and Informatics, University of Heidelberg, Im Neuenheimer Feld 305, Heidelberg, 69120, Germany; 6 Department of General Surgery, Klinikum rechts der Isar, Ismaninger Straße 22, München, 81675, Germany

## Abstract

A response to Seiler et al: Interrupted or continuous slowly absorbable sutures for closure of primary elective midline abdominal incisions: a multicenter randomized trial (INSECT: ISRCTN24023541). Ann Surg 2009, 249(4):576-582.

**Background:**

Existing evidence suggests that the transfer of results of randomized controlled trials into clinical practice may be limited. Potential reasons can be attributed to aspects of external validity. The aim of this study is to investigate issues related to the external validity of the INSECT trial.

**Methods:**

All participating surgical departments were categorized and the clinical and baseline characteristics of randomized patients were evaluated. In addition, demographic and clinical data of all screened and randomized patients at the Departments of Surgery in Heidelberg and Erlangen were analyzed.

**Results:**

Twenty-five centers enrolled a total of 625 patients. These centers included eight primary, 11 secondary, and six tertiary care centers. The tertiary care centers enrolled the most patients (n = 237, 38%) followed by the primary care centers (n = 199, 32%) and the secondary care centers (n = 189 patients; 30%). The mean number and baseline data of randomized patients did not differ between the three types of care centers (p = 0.09). Overall, the treatment according to protocol was at least 92%. At the Department of Surgery, University of Heidelberg, 307 patients were screened and 60 out of 130 eligible patients were randomized. There were no differences in demographic and clinical baseline data between included and non-included patients. In Erlangen, 351 patients were screened and 57 out of 106 eligible patients randomized.

**Conclusions:**

Results of the INSECT trial are applicable to a broad spectrum of patients treated at different hospital levels.

## Background

Incisional hernias of midline incisions are the most common long-term complication after major abdominal surgery with an incidence ranging from 5% - 24% [1]. Although randomized clinical trials (RCTs) and meta-analyses [2] have been performed in order to define optimal closure strategies, clinical uncertainty still has not been resolved.

It has been shown that RCTs have average patient exclusion rates ranging from 73% to 97% [3]. This "selection" of patients together with the assumption that patients enrolled in RCTs receive potentially better treatment than their non-included counterparts [4] may weaken the transfer of obtained results into routine practice. In general, factors which potentially impair the clinical translation of results achieved in RCTs are summarized under the terms of internal and external validity. Internal validity of RCTs focuses on accuracy and precision in order to minimize potential biases and chance [5]. According to Rothwell [3], issues that potentially affect external validity can be summarized as setting of the trial, selection of patients, characteristics of randomized patients, differences between the trial protocol and routine practice, outcome measures and follow-up examinations, as well as adverse effects of treatments (table 1). In addition, accepted definitions which relate to how representative a study population is were determined (e.g., eligibility fraction, enrollment fraction, recruitment fraction) [6].

**Table 1:** Issues that potentially affect external validity adapted to Rothwell (10).

Heading	
**Setting of the trial**	

	Healthcare system

	Country

	Recruitment from primary, secondary, or tertiary care

	Selection of participating centers

	Selection of participating clinicians

**Selection of patients**	

	Eligibility criteria

	Exclusion criteria

	Random ratio

	Patients declining randomization

**Characteristics of randomized patients**	

	Baseline clinical characteristics

	Severity of disease

	Comorbidity

**Differences between trial protocol and routine praxis**	

	Trial intervention

	Therapeutic or diagnostic advances since the trial was completed

**Outcome measures and follow-up**	

	Who measured outcome

	Frequency of follow-up

	Adequate length of follow-up

**Adverse effects of treatment**	

	Completeness of reporting

	Selection of trial centers/clinicians

	Intensity of trial safety procedures

The internationally registered INSECT trial was designed and conducted as a multicenter RCT (mRCT) comparing two continuous suture techniques with slowly absorbable monofilament materials (PDS™ or MonoPlus™) and one interrupted suture (Vicryl™) for fascial closure after primary elective midline incisions [1]. The primary endpoint of the INSECT trial was the occurrence of incisional hernias within one year after the surgical intervention. The trial was conducted on 625 randomized patients and its primary analysis showed no differences in the occurrence of incisional hernias between the three groups after one year. Further, there were no significant differences with regard to burst abdomen, wound infection, pulmonary infections, serious adverse events, and one-year mortality [1]. The objective of this analysis was to investigate issues of external validity of the INSECT trial focusing in particular on institutional and selection biases.

## Methods

During the INSECT trial, 625 patients were randomized at 25 surgical sites between July 11, 2004, and September 26, 2006. The detailed study protocol of the INSECT trial describes several strategies to ensure high internal validity including primary hypothesis, randomization, sample size calculation, harmonization and standardization of treatment, assessment and bias [1]. In addition, issues that potentially affect external validity were recorded, namely detailed information describing the process from screening to randomization in two participating centers.

### Setting of the trial

The types of all participating surgical departments were categorized into primary, secondary or tertiary care centers. Primary care centers (PCC) were defined as hospitals with basic diagnostic and therapeutic options offering general surgical interventions. Secondary care centers (SCC) were hospitals with a broader surgical spectrum (i.e. more specialized departments) and advanced diagnostic and therapeutic tools to which a patient had been referred by primary care providers. Tertiary care centers (TCC) act as referral centers and are often academic hospitals with the highest standards of care, including access to most specialists and the necessary equipment that may be lacking in primary and secondary care centers. The selection of participating centers and surgeons was purely based on the self-motivation of the according centers. Once they agreed to adhere to the protocol, signed a contract, received ethical approval from their local ethics committee and finalized training, they were able to start patient recruitment. In order to assess whether this process caused imbalances between the patient populations at the different hospital levels, analyses concerning the recruitment of patients and demographics (i.e. age and gender) as well as clinical parameters (body mass index) and surgery-related parameters (type of procedures) were performed. In addition, the level of expertise of surgeons and the adherence to the protocol were assessed.

### Selection of patients

Inclusion and exclusion criteria of the trial were shown previously [1]. Due to the incomplete recording of screening lists, the according data was not available from other participating centers. Patients at the Department of Heidelberg were analyzed for demographics (age and gender), clinical parameters (body mass index, ASA score, duration of hospitalization, presence of malignant diseases), and surgery-related parameters (incision technique, closure technique) according to three specified subgroups: included patients, eligible patients who declined participation and eligible patients randomized to one other RCT.

The random ratio was calculated by dividing the number of randomized patients/participants by the number of primarily eligible patients/eligible for participation for the Department of Surgery of the University of Heidelberg and Erlangen. The random ratio thus equals the enrollment fraction as described by Gross et al. [6]. According to Gross et al. [6] and Jones et al. [7], the enrollment fraction equals the proportion of people who are eligible for participation and who actually enroll in the RCT.

Data analysis was done using SAS™ 9.1 Win (Release 9.1, SAS Institute, Inc., Cary, NC). A description of the data included absolute and relative frequencies for categorical data and mean and standard deviations for continuous data. Possible differences between groups were calculated with Fisher's exact test of categorical parameters, Kruskal-Wallis test for continuous variables, and binomial test for proportion of randomized patients. The explorative statistical significance level was set at 5% (0.05); no adjustment for multiple comparisons was done due to the descriptive nature of the study.

This article was structured based on the recommendations according to the Strengthening the Reporting of Observational Studies in Epidemiology (STROBE) in order to include an accurate and complete report of an observational study [8]. The original INSECT trial was published in accordance with the CONSORT criteria [1].

## Results

### Setting of the trial

**Figure 1:**
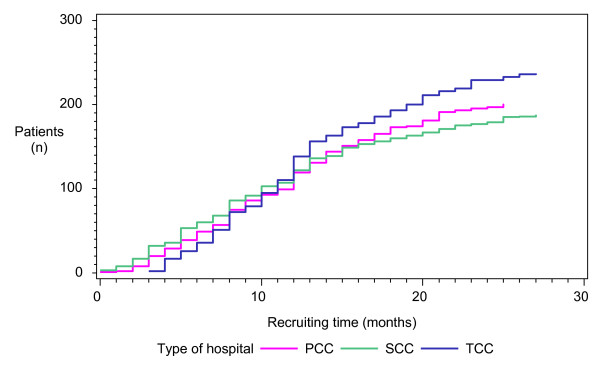
**Recruiting of 625 patients during the INSECT trial according to PCC, SCC and TCC, respectively**.

**Figure 2:**
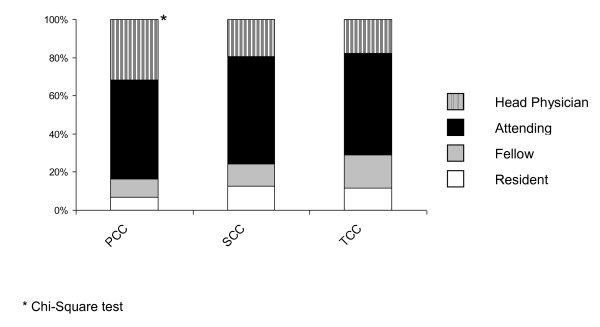
**Educational status of surgeon who performed the surgical procedures in PCC, SCC and TCC**.

The INSECT trial was conducted in Germany. Centers were recruited on a voluntary basis. Overall, twenty-five centers enrolled a total of 625 patients between July 11, 2004, and September 26, 2006 (table 2). The distribution of hospitals according to the three categories was as follows: 8 PCC, 11 SCC and 6 TCC. TCC enrolled more patients (n = 237, 37.92%) than PCC (n = 199, 31.84%) and SCC (n = 189 patients; 30.24%). There were no differences in the recruiting of patients between the three different types of hospitals over time (Figure 1). In order to test whether the number of randomized patients per category differed from normal distribution (i.e. the assumption that each hospital category enrolled one-third, or 33.3% of all patients), a binomial test was performed. Even though the percentage of randomized patients to each of the three hospital types (PCC, SCC, TCC) differed significantly (p < 0.01, Kruskal-Wallis test, table 2), the observed differences of mean randomized patients per hospital type were not different (p = 0.09, Kruskal-Wallis test, table 2). Significantly more patients were operated on by head physicians at PCC compared to SCC and TCC (p < 0.01, Chi-Square test, Figure 2).

**Table 2:** Analysis of participating centers.

Type of care center	n	Randomized patients (n)	Percentage of randomized patients (%)*	Mean of randomized patients (Standard Deviation) #
**PCC**	8	199	31.84	25.0 (12.9)

**SCC**	11	189	30.24	16.5 (14.0)

**TCC**	6	237	37.92	39.3 (24.1)

**Sum**	25	625	100	-

* p < 0.01, Kruskal-Wallis test

# p = 0.09, Kruskal-Wallis test

### Selection of patients

During the recruitment phase of the INSECT trial, all patients admitted with a diagnosis requiring a midline laparotomy were screened to be randomized to the INSECT trial. After controlling all inclusion and exclusion criteria and receiving informed consent, patients were included for randomization.

Overall, 625 patients were randomized (37.8% female patients). Analyzing all randomized patients based on age, gender, and body mass index, there were no differences between PCC, SCC and TCC. Patients with tumors located at either the stomach/esophagus or pancreas were significantly more often operated on at SCC and TCC whereas there were no differences in colorectal procedures (table 3). Significantly more patients were treated according to protocol in PCC and SCC as compared to TCC (table 3). The number of patients lost to follow-up did not differ significantly between PCC, SCC and TCC (table 3).

**Table 3:** Baseline characteristics of all randomized patients.

Parameter	PCC	SCC	TCC	p-value (Chi-Square)
**Age (years)**				

Mean (SD)	67.7 (10.8)	64.7 (11.5)	61.3 (14.2)	n.s.

**Gender (n)**				

female	70	73	86	n.s.

male	118	107	151	n.s.

**Body Mass Index (kg/m^2^)**				

Mean (SD)	26.2 (3.7)	25.9 (3.9)	25.6 (3.7)	n.s.

**Surgical Procedure (n)**				

vascular	3	3	15	< 0.01

large intestine	106	85	82	n.s.

small intestine	1	0	7	< 0.01

rectum	68	40	37	n.s.

stomach/oesophagus	8	44	28	< 0.01

pancreas	2	2	40	< 0.01

**Treatment according to protocol (%)**				

yes	98.4	97.2	92.4	< 0.01

**Lost to follow-up (%)**				

	37.5	38.3	28.7	0.06

At the Department of Surgery, University of Heidelberg, 307 patients scheduled for elective abdominal surgery were screened at admission (24 hours before surgery) for eligibility from January 13 to August 25, 2005. At the Department of Surgery, University of Erlangen, 351 patients were screened between October 21, 2004, and February 24, 2006.

**Figure 3:**
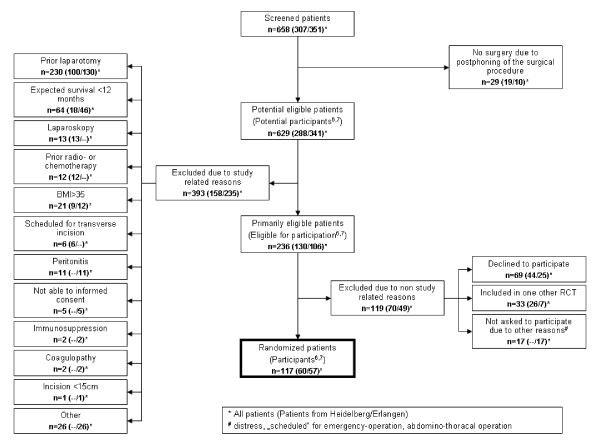
**Depiction of the recruitment process at the Departments of Surgery in Heidelberg and Erlangen**.

The flow chart of patients at both institutions with corresponding numbers is given in Figure 3. Altogether, 29 out of these 658 patients were excluded because of new findings during the preoperatively performed diagnostic work-up. Out of the remaining 629 patients (100%), 236 patients (37.5%) were primarily eligible to participate in the INSECT trial and 393 patients (62.5%) had to be excluded for not fulfilling all inclusion criteria or for meeting one or more exclusion criteria. Most of these patients (n = 230) were excluded because of prior laparotomy. A further 119 patients who were considered primarily eligible had to be excluded due to non-study related reasons; 69 patients declined randomization; and 33 patients participated in one other RCT [9]. Subsequently, a total of 117 patients (18.6% of 629 potential eligible patients) were randomized into the INSECT trial resulting in an random ratio (equals enrollment fraction [6,7] of 49.5% (Heidelberg 46.1%, Erlangen 53.7%).

Based on the given data, the overall eligibility fraction was 37.5% (Heidelberg 45.1%, Erlangen 31.1%) and the overall recruitment fraction was 18.6% (Heidelberg 20.8%, Erlangen 16.7%).

### Characteristics of randomized patients

From the 625 randomized patients, there were 210 patients randomized to interrupted suture using an absorbable braided suture material, 205 patients randomized to continuous suture techniques using PDS™, and 210 patients randomized to continuous suture techniques using MonoPlus™. All 625 patients were of Caucasian origin. After analyzing demographics (age and gender), clinical parameters (body mass index), and surgery-related parameters (surgical procedures) of all randomized patients, there were no differences between the three randomized groups (table 3). The indication for the surgical procedures did not differ between the three hospital types. In 83% of PCC, 82% of SCC, and 83% of TCC, the primary diagnosis was associated with the rectum, colon, and stomach/esophagus. Further, the incision length did not differ significantly between the randomized groups whereas the closure times for the continuous groups were significantly shorter compared to the interrupted group (P < 0.01; [1]).

### Surgeons training for standardizing abdominal wall closure

Midline incision of the abdominal wall is a preferred strategy to open the abdominal cavity. However, there is no consent within the surgical community concerning the optimal abdominal wall closure after midline incision. For the INSECT trial, the two most often used methods of abdominal wall closure were compared using three different suture materials. In order to harmonize the closure techniques between the different centers and participating surgeons as much as possible, all participating centers were trained in how to perform the different suturing techniques adequately before randomization of the first patient. The results for the three different hospital categories were excellent with more than 90% of all patients treated according to protocol.

### Outcome measures and follow-up examinations

Patients were observed up to 30 days after the primary surgical intervention to record adverse and serious adverse events and early complications defined as secondary endpoints (burst abdomen, wound infection, and postoperative pulmonary complications). The definition for secondary endpoints is published elsewhere [1]. The 30-day follow-up was conducted by coordinating personnel. The frequency of incisional hernias was examined after one year. A surgeon performed the clinical examination and at minimum an ultrasound of the abdominal wall had to be performed by a radiologist. Coordinating personnel as well as surgeons and radiologists involved in follow-up examinations were blinded to the method of closing the abdominal wall.

### Adverse effects of treatments

During the INSECT trial, adverse events were monitored and classified by an adverse events committee (AEC) consisting of three surgeons. All information on adverse events were initially documented and verified by coordination personnel and eventually submitted to the AEC. All adverse events were defined in the published protocol [1]. All serious adverse events were reported to the according regional councils.

## Discussion

External validity describes to which extent the results of RCTs are applicable to patients treated in general practice. Together with the primary publication [1], the focus of this analysis was to investigate further aspects relevant for the external validity of the INSECT trial mainly according to the issues raised by Rothwell [10]. As the data presented here comprises a significant amount of new information, it seemed reasonable to publish this data as a separate article.

Because of prospective data acquisition, external monitoring and external data analysis the INSECT trail is an optimal source for analyzing external validity. The INSECT trial was a multicenter German RCT. Thus, there were no differences based on different healthcare systems or countries. The participation was voluntarily suggesting that only motivated surgeons got involved. Even though the number of participating TCC was the lowest, the mean number of randomized patients did not differ between PCC, SCC and TCC, respectively.

One main conclusion of this paper would be that smaller hospitals such as PCC (or SCC) should be involved in clinical trials whenever possible because these hospitals are important resources in terms of patient recruitment. Not only that, but the here presented analyses also revealed that the randomization process occurred at similar speeds in all three types of hospitals; the adherence to the protocol was significantly better in PCC and SCC; the number of included patients and the main characteristics of included patients did not differ between the three hospital types; and the number of patients lost to follow-up did not differ significantly between PCC, SCC and TCC. Thus, the message of the INSECT trial (i.e. the rate of incisional hernias after midline incision is not dependent on the used suture material/technique) is truly independent of the hospital type in which the patient is operated.

Reasons for not participating in a surgical study are multifactorial and were not assessed systematically within this study. However, they include well-known reasons such as the dislike of randomization procedures, strict protocols for performance of interventions and unwillingness for follow-up investigations. In order to detect potential selection bias, the screening processes at the Departments of Surgery at the Universities of Heidelberg and Erlangen were assessed in detail. The process from screening until randomization could be described in detail in 116 patients out of 625 randomized patients (18.5%). Even though this alone may not allow us to draw general conclusions about external validity, other parameters such as the progress of randomized patients between the different hospitals and the clinical data of included patients were also similar. This suggests that the process from screening to randomization may be also comparable in all participating centers.

The largest group of excluded patients had a prior laparotomy. Because this patient group may not benefit from the results achieved during the INSECT trial, further studies are needed to define the optimal method of abdominal wall closure for patients with prior laparotomy. Out of the remaining potentially eligible patients, another 119 patients had to be excluded because of two reasons: firstly, patients refused randomization; and secondly, patients were already included in another RCT, one of which was the POVATI trial [9].

Compared to the multicenter INSECT trial, the POVATI trial was conducted as a single center trial at the Department of Surgery, University of Heidelberg. Given the inclusion and exclusion criteria, patients with primary laparotomy were eligible to participate in both RCTs. The decision by patients to participate in one or the other study could have an impact on external validity. Because there were no differences in demographics and baseline characteristics between patients included in either trial, it is unlikely that patients enrolled in the POVATI trial will affect the external validity of the INSECT trial (table 4). The comparison of the 130 potential eligible patients in Heidelberg revealed significant differences only in the applied incision and closure techniques in non-consenting patients (n = 44) and patients randomized to the POVATI trial (n = 26). For non-consenting patients, surgeons could choose freely their preferred techniques for opening and closing the abdomen. When given the choice, the vast majority of surgeons at our hospital chose midline laparotomy as the primary abdominal approach and continuous suturing as preferred closure technique. This may suggest that surgeons at our hospital were somehow biased towards midline laparotomy and continuous suturing (table 4).

**Table 4:** Baseline characteristics of the 60 included patients compared to the 70 patients who had to be excluded due to non-study related reasons.

Parameter	Patients in INSECT trial (n = 60)	Patients not consenting (n = 44)	Patients in POVATI trial (n = 26)	p-value
**Age**				

Mean (SD)	57.70 (17.08)	55.93 (17.54)	56.69 (12.27)	0.6088*

**Gender**				

female	21 (35.00%)	17 (38.64%)	9 (34.62%)	

male	39 (65.00%)	27 (61.36%)	17 (65.38%)	

				0.9158^#^

**Body Mass Index**				

Mean (SD)	25.62 (3.41)	24.61 (3.60)	23.77 (2.51)	0.0786*

**ASA score**				

I	4 (6.67%)	2 (4.55%)	2 (7.69%)	

II	34 (56.67%)	27 (61.36%)	15 (57.69%)	

III	21 (35.00%)	15 (34.09%)	9 (34.62%)	

IV	1 (1.67%)	0 (0.00%)	0 (0.00%)	

				0.9931^#^

**Duration of hospitalization**				

Mean (SD)	14.61 (11.76)	14.34 (11.64)	13.00(6.90)	0.9570*

**Malignant disease**				

none	35 (58.33%)	19 (43.18%)	10 (38.46%)	

solid tumors	24 (40.00%)	25 (56.82%)	16 (61.54%)	

missing/n.a.	1 (1.67%)	0 (0.00%)	0 (0.00%)	

				0.1634^#^

**Incision technique**				

midline	60 (100.00%)	40 (90.91%)	18 (69.23%)	

transverse	0 (0.00%)	3 (6.82%)	8 (30.77%)	

laparoscopic	0 (0.00%)	1 (2.27%)	0 (0.00%)	

				0.0001^#^

**Closure technique**				

continuous suture	40 (66.67%)	39 (88.64%)	26 (100.00%)	

interrupted suture	20 (33.33%)	4 (9.09%)	0 (0.00%)	

missing/n.a.	0 (0.00%)	1 (2.27%)	0 (0.00%)	

				0.0006^#^

* Kruskal-Wallis test, ^# ^Fisher's exact test

In general, the random ratio ("enrollment fraction" according to Gross et al.) describes the proportion of randomized patients to the primarily eligible patients. The random ratio per se does not say anything as to whether the results of a study are widely acceptable. It is more likely to be a marker of the efforts necessary to randomize a patient. Even though the random ratio at the Department of Surgery at the University of Erlangen was similar to that at the University of Erlangen, the recruitment period lasted longer in Erlangen (16 months versus 6 months at Heidelberg). One possible explanation could be that all surgical trainees in Heidelberg rotate into the clinical study centre and residents who already finished their rotation are part of an on-call program. This setting allows screening and inclusion of patients even at night or on weekends. In contrast, one clinical investigator was primarily responsible for patient recruitment in Erlangen. Considering missing days due to holidays, weekends, days off after being on call, etc., screening and recruiting of patients was only possible on 120 days.

## Conclusion

Most of the criteria for external validity as suggested by Rothwell [10] have been fulfilled by the INSECT trial.

## Abbreviations

INSECT: **I**nterrupted or co**n**tinuous slowly absorbable **s**uture - Design of a multi-center randomized trial to **e**valuate abdominal **c**losure **t**echniques; PCC: Primary care center; RCT: Randomized controlled trial; SCC: Secondary care center; TCC: Tertiary care center

## Competing interests

Hanns-Peter Knaebel is currently working as CEO of Aesculap AG, GmbH, Tuttlingen, which was one of the sponsors of this study. Other than that there are no competing interests to state.

## Authors' contributions

Study planning: HPK, CMS, MWB, LF, JB, TB; Data collection: HG, LF, HPK, MD, JB, CMS; Data analysis: TB, MD, CMS, LF; Writing of article: LF, HG, JB; Critical revision: MWB, CMS, HPK; Final approval: all authors

## Pre-publication history

The pre-publication history for this paper can be accessed here:

http://www.biomedcentral.com/1471-2482/12/2/prepub
